# Secapin: a promising antimicrobial peptide against multidrug-resistant Acinetobacter baumannii

**DOI:** 10.3205/dgkh000491

**Published:** 2024-07-09

**Authors:** Zohreh Sadeghi Rad, Mahnaz Farahmand, Mahsa Kavousi

**Affiliations:** 1 Department of Biology, East Tehran Branch, Islamic Azad University, Tehran, Iran

**Keywords:** Acinetobacter baumannii, antimicrobial, Secapin, bactericidal, antibiofilm, cell compatibility

## Abstract

**Introduction:**

*Acinetobacter baumannii*, renowned for its exceptional multidrug resistance and its role as a prevalent nosocomial pathogen, poses a formidable challenge to conventional antibiotic therapies. The primary objective of this investigation was to evaluate the efficacy of Secapin, an antimicrobial peptide, against multidrug-resistant (MDR) *baumannii*. Furthermore, the mechanisms underlying Secapin’s antibacterial and antibiofilm activities were elucidated.

**Methods:**

The antimicrobial and antibiofilm effectiveness of Secapin against MDR *A. baumannii* was assessed through a series of experiments. The minimum inhibitory concentration (MIC) and minimum bactericidal concentration (MBC) of Secapin were determined using established protocols. Time-kill kinetic analysis was performed to assess the concentration-dependent bactericidal effect of Secapin. Additionally, the capacity of Secapin to impede biofilm formation and eradicate *A. baumannii* biofilms was investigated. Hemolytic potential was evaluated using human red blood cells, while mammalian cell viability was examined at varying Secapin concentrations.

**Results:**

Secapin exhibited robust bactericidal activity at minimal concentrations, with an MIC of 5 µg/mL and an MBC of 10 µg/mL against MDR *A. baumannii*. The time-kill kinetic analysis confirmed the concentration-dependent efficacy of Secapin in diminishing bacterial viability. Moreover, Secapin demonstrated the ability to prevent biofilm formation and eliminate established *A. baumannii* biofilms. Notably, Secapin exhibited no hemolytic activity and preserved mammalian cell viability up to a concentration of 100 µg/mL.

**Conclusion:**

These findings underscore the substantial potential of Secapin as a potent agent against multidrug-resistant *A. baumannii*, showcasing its efficacy in both antibacterial and antibiofilm capacities. The favorable attributes of Secapin, characterized by its minimal hemolytic effects and high mammalian cell viability, position it as a promising contender in the fight against antibiotic resistance.

## Zusammenfassung

**Hintergrund:**

*Acinetobacter baumannii*, bekannt für seine außergewöhnliche Multiresistenz und seine Rolle als häufiger nosokomialer Erreger, stellt eine Herausforderung für herkömmliche Antibiotikabehandlungen dar. Das Hauptziel dieser Studie war es, die Wirksamkeit von Secapin, einem antimikrobiellen Peptid, gegen multiresistente *A. baumannii *zu bewerten. Darüber hinaus wurden die Mechanismen, durch die Secapin seine antibakterielle und antibiofilmbildende Aktivitäten entfaltet, aufgeklärt.

**Methoden:**

Die antimikrobielle und antibiofilmbildende Wirksamkeit von Secapin gegen MDR *A. baumannii* wurde in einer Reihe von Experimenten untersucht. Die minimale Hemmkonzentration (MIC) und die minimale bakterizide Konzentration (MBC) von Secapin wurden unter Verwendung etablierter Protokolle bestimmt. Es wurde eine zeitabhängige Abtötungskinetik ermittelt, um die konzentrationsabhängige bakterizide Wirkung von Secapin zu bewerten. Darüber hinaus wurde die Fähigkeit von Secapin, die Bildung von Biofilmen zu hemmen und etablierte *A. baumannii*-Biofilme zu beseitigen, untersucht. Das hämolytische Potenzial wurde unter Verwendung von menschlichen roten Blutkörperchen bewertet, während die Lebensfähigkeit von Säugetierzellen bei verschiedenen Secapin-Konzentrationen untersucht wurde.

**Ergebnisse:**

Secapin zeigte eine starke bakterizide Aktivität bei geringen Konzentrationen mit einer MIC von 5 µg/mL und einer MBC von 10 µg/mL gegen MDR *A. baumannii*. Die zeitabhängige Abtötungskinetik bestätigte die konzentrationsabhängige Wirksamkeit von Secapin. Darüber hinaus zeigte Secapin die Fähigkeit, die Bildung von Biofilmen zu verhindern und etablierte *A. baumannii*-Biofilme zu eliminieren. Bemerkenswert ist, dass Secapin keine hämolytische Aktivität aufwies und die Lebensfähigkeit von Säugetierzellen bis zu einer Konzentration von 100 µg/mL aufrechterhielt.

**Fazit:**

Die Ergebnisse unterstreichen das erhebliche Potenzial von Secapin als wirksames Mittel gegen multiresistente *A. baumannii* sowohl in Bezug auf seine antibakterielle als auch antibiofilmbildende Aktivität. Die vorteilhaften Eigenschaften von Secapin, gekennzeichnet durch seine geringen hämolytischen Effekte und hohe Verträglichkeit gegenüber Säugetierzellen, positionieren es als vielversprechenden Kandidaten im Kampf gegen Antibiotikaresistenzen.

## Introduction

*Acinetobacter baumannii*, a Gram-negative, non-motile, strictly aerobic coccobacillus, is a pivotal member of the Acinetobacter complex, encompassing *A. baumannii*, *A. calcoaceticus*, and genomic species 13TU [1]. Renowned for its high transmissibility, *A. baumannii* is implicated in a spectrum of infections affecting diverse human organs, leading to complications such as pneumonia, meningitis, septicemia, urinary tract infections, and abscesses. Transmission of this pathogen can occur through direct contact or exposure to contaminated sources like water, food, and soil, particularly affecting individuals with prolonged hospital stays, thus classifying it as a nosocomial pathogen [2]. The emergence of antibiotic-resistant strains of* A. baumannii* poses a significant challenge, showcasing resistance to colistin, aminoglycosides, β-lactams, and tetracycline through mechanisms including β-lactamase acquisition, up-regulation of multidrug efflux pumps, aminoglycoside modification, permeability defects, and target site alterations [3]. Additionally, *A. baumannii* is a member of the ESKAPE pathogens, a group of high-risk infectious agents responsible for healthcare-associated infections, further complicating treatment strategies.The escalating difficulty in finding effective and readily available antibiotics emphasizes the urgent need for innovative therapeutic agents against ESKAPE species, highlighting the critical importance of addressing the challenges posed by multidrug-resistant *A. baumannii* in the clinical setting [4].

Bee venom represents a complex blend of bioactive compounds, including polypeptides, enzymes, amines, lipids, and amino acids, as supported by multiple research studies [5]. While the allergenic nature of bee venom constituents is well-documented, they are also utilized in traditional and complementary medicine for their anti-arthritis, analgesic, and anti-inflammatory properties [6]. 

Secapin, a peptide component derived from bee venom, was first discovered approximately four decades ago. While studies have shown that Secapin is non-toxic in murine models, high doses have been associated with effects like sedation, piloerection, and hypothermia [7]. Subsequent research efforts have led to the identification of the complete cDNA sequence encoding preprosecapin, with mature secapin consisting of 25 amino acid residues featuring a disulfide bond. Despite being acknowledged as a potent neurotoxin, the full spectrum of functions attributed to secapin remains largely unexplored. Recent research has revealed diverse attributes of secapin, encompassing hyperalgesic and edematogenic effects, with limited studies focusing on its antibacterial effects [8].

In this study, a series of experiments was conducted to evaluate the efficacy of Secapin against multidrug-resistant (MDR) *A. baumannii* infections in vitro, with a particular emphasis on its antibacterial properties.The antibiotic resistance profile of *A. baumannii* strain was initially characterized in this study. Following this, the minimum inhibitory concentration (MIC) and minimum bactericidal concentration (MBC) of Secapin were determined to assess its antibacterial efficacy. Furthermore, the toxicity profile of Secapin was investigated using haemolysis and cell viability assays in mammalian cells. 

## Materials and methods

### Preparation of Scapin

Secapin, with the chemical formula C131H213N37O31S2 and CAS number 58694-50-1, was sourced from Hangzhou Jhechem Co., Ltd., for use in our microbiology research studies. 

### Growth condition and confirmation of strain

The *A. baumannii* strain (Number 37) utilized in this study was isolated from hospitalized ICU patient subsequently verified through 16S rRNA sequencing conducted by Bionics (Seoul, Korea) using universal primers. Authentication was achieved using the 27F forward primer (5’-AGAGTTTGATYMTGGCTCAG-3’) and 1525R reverse primer (5’-AGAAAGGAGGTGATCCAGCC-3’) [9]. Throughout all experimental procedures, *A. baumannii* cultures were maintained at a bacterial cell density of 1x10^6^ colony forming units (CFU)/mL, corresponding to an optical density at 595 nm of 0.08, unless specified otherwise. In this investigation, *Acinetobacter baumannii* ATCC 19606 was utilized as the model reference strain.

### Determination antimicrobial susceptibilityof A. baumannii

The antibiotic susceptibility profile of *Acinetobacter baumannii* was evaluated through the antibiotic disc diffusion method, employing 17 antibiotics representing diverse classes sourced from Mast Diagnostic Group (UK). In brief, *A. baumannii* culture was evenly distributed on Muller Hinton Agar (MHA) plates, followed by aseptic placement of antibiotic discs on the agar surface and subsequent incubation at 25°C for 24 hours. The diameter of the zone of inhibition surrounding the disc (6 mm) was then carefully measured. Antibiotic susceptibility of the *A. baumannii* strain was classified as resistant, intermediate, or susceptible by analyzing the observed zone sizes with the standard zone diameter interpretation chart.

### Determination of the time-kill kinetics, MIC, and MBC of Secapin for A. baumannii

The time-kill dynamics, minimum inhibitory concentration (MIC) and minimum bactericidal concentration (MBC) were assessed using a microdilution susceptibility test for MIC and the agar plating method for MBC, in accordance with Clinical and Laboratory Standards Institute (CLSI) M07-A guidelines. *A. baumannii* cultures at a concentration of 1x10^6^ CFU/m) were added to triplicate wells of a 96-well microplate (190 µL/well). Varying concentrations (ranging from 0 to 20 µg/mL) of Secapin were then added in 10 µL aliquots to each well, while chloramphenicol, ranging from 0 to 100 µg/mL, served as positive control. After incubation for 24 hours at 25°C, bacterial proliferation was quantified at 3-hour intervals (0, 3, 6, 9, 12, 18 and 21 hours) by measuring optical density at 595 nm using a microplate spectrophotometer (Bio-Rad, Saint Louis, MO, USA) for time-kill kinetics analysis. The minimum concentration showing no discernible change in optical density (indicating no visible growth) after 24 hours was designated as the MIC. MBC was determined by plating 100 µL aliquots of bacterial suspensions from the MIC test wells, containing peptide concentrations equal to or greater than the MIC values, onto tryptic soy agar (TSA) plates. The lowest concentration that did not produce bacterial colonies after 24 hours of incubation at 25°C was identified as the MBC.

### Determining the viability of Acinetobacter baumannii cells after Secapin treatment

The MTT assay for Secapin-treated *A. baumannii* was performed according to the method described by Dananjaya et al. [9] 2 ml of *A. baumannii* culture (1x10^6^ CFU/ml) was treated with different concentrations (0–10 g/ml) of Secapin and chloramphenicol (50 g/ml) as positive control. After incubation at 25°C for 24 hours, samples were centrifuged at 1,500 g for 10 min and cells were washed with PBS. Thereafter cells were washed with 20 L of 5 g/mL MTT reagent (Sigma Aldrich, Munich, Germany) for 30 min. Dimethyl sulfoxide (DMSO) (Sigma Aldrich, Munich, Germany) was then added to the samples and the cells were resuspended. OD595 was measured using a microplate spectrophotometer.

### Effect of Secapin on A. baumannii biofilm formation inhibition and eradication

To investigate the effect of Secapin on the inhibition and eradication of *A. baumannii* biofilm formation, we performed antibiofilm assays using crystal violet (CV) staining as described by Kim et al. [10]. For the biofilm inhibition assay, *A. baumannii* (10^6^ CFU/mL) in tryptic soy broth (TSB) supplemented with 0.2% glucose was added to a 96-well plate containing Secapin (10 L) at various final concentrations (0–12.5 g/mL) and incubated at 25°C for 24 hours. In the biofilm eradication assay, *A. baumannii* (10^6^ CFU/mL) in TSB supplemented with 0.2% glucose was added to a 96-well plate (100 L/well) and the plate was incubated at 25°C for 24 hours until biofilm formation occurred. The supernatant was then removed and the walls carefully washed with PBS. TSB supplemented with 2% glucose was then added to each well and treated with Secapin (0–12.5 g/mL). The plate was then incubated at 25°C for 24 hours. The remaining biofilms in both the inhibition and eradication assays were quantified using the CV technique. The supernatant containing planktonic bacteria was removed and the walls washed with PBS. Subsequently, 100% methanol was added to the wells to fix the biofilm, which was washed after 10 minutes. The biofilms were then stained with a 0.1% (w/v) solution of CV (Sigma-Aldrich, Munich, Germany) for 30 minutes. Excess CV was removed by three washes with PBS. Finally, the biofilm was completely dissolved in 95% ethanol and the absorbance was measured at a wavelength of 595 nm using a microplate spectrophotometer. The quantification of the inhibitory effect on the formation of the biofilm was conducted by employing Equation 1: 

Biofilm formation inhibition/eradication %=(1–(Ab test^1^)*100%

(Ab negative control^2^)

^1^Absorbance value of the Secapin- or chloramphenicol-treated test group

^2^Absorbance value of the negative control

### Hemolysis assay of Secapin

A haemolysis experiment was performed on Secapin-treated mouse red blood cells (RBCs) according to the protocol described by Kim et al. [10]. RBCs were washed thoroughly and suspended in PBS. Secapin was then added at a concentration range of 0–500 g/mL.1% (v/v) Triton X-100 (Sigma Aldrich, Munich, Germany) was added as positive control. PBS was used as negative control. After one hour of incubation at room temperature, the supernatant was separated by centrifugation. The absorbance of the supernatant (200 L) was measured at 415 nm using a microplate spectrophotometer (Bio-Rad, Saint Louis, MO, USA). The percentage of RBC hemolysis was determined using Equation 2:


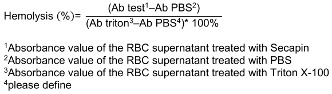


### Cytotoxicity of Secapin against murine macrophage cells

The MTT assay was performed on Secapin-treated RAW 264.7 murine cells. These cells were cultured in Dulbecco’s modified Eagle’s medium (Sigma-Aldrich, Munich, Germany) supplemented with 10% (v/v) fetal bovine serum (Sigma-Aldrich, Munich, Germany) and an antibiotic-antimycotic solution (Thermo Fisher Scientific, Waltham, MA, USA) and maintained at 37°C in a humidified atmosphere with 5% CO_2_ for 24 hours. The cells were then seeded at a density of 2x10^5^ cells/mL (100 µL/well) into a 96-well flat-bottomed microtiter plate and allowed to adhere for 12 hours. The culture medium was then replaced with varying concentrations of Secapin (ranging from 20 to 500 µg/mL) together with 10 µL of PBS as negative control and incubated under the same conditions for 24 hours. The culture medium was then replaced with 90 µL of fresh medium and 10 µL of 5 µg/mL MTT (Sigma-Aldrich, Munich, Germany) was added to each well, followed by incubation at 37°C for 4 hours. After the incubation period, the culture medium was aspirated and 50 µL DMSO (Sigma-Aldrich, Munich, Germany) was added to dissolve the formazan dye. The absorbance was then measured at 595 nm using a microplate spectrophotometer.

## Results

### Isolation and molecular confirmation of A. baumannii

The nucleotide BLAST analysis effectively delineated the taxonomic classification of our isolate to the species level.

### Antibiotic susceptibility of A. baumannii

Among the 18 antibiotics tested, a substantial percentage (55.5%), such as gentamycin, streptomycin, vancomycin, cefotaxime, penicillin, erythromycin, clindamycin, tobramycin, trimethoprim, and rifampin, demonstrated resistance against *A. baumannii*. Notably, vancomycin, penicillin, erythromycin, clindamycin, trimethoprim and rifampin showed zero zones of inhibition. In addition, tetracycline, amikacin, ciprofloxacin and sulfamethoxazole/trimethoprim showed intermediate resistance (22.2%). Conversely, only three antibiotics (chloramphenicol, ofloxacin, and doxycycline) showed susceptibility (22.2%) and resulted in complete inhibition of *A. baumannii* growth (Table 1 (Tab. 1)).

### Minimum inhibitory concentration and minimum bactericidal concentration of A. baumannii exposed to Secapin

Secapin exhibited strong antibacterial activity with an MIC of 5 g/mL and an MBC of 10 g/mL, giving an MBC/MIC ratio of 2.0. The standard antibiotic chloramphenicol (used as positive control) also showed antibacterial activity with MIC and MBC values of 10 g/mL and 50 g/mL respectively.

### Time-kill kinetics and bacterial viability of A. baumannii upon Secapin

**Figure 1:**
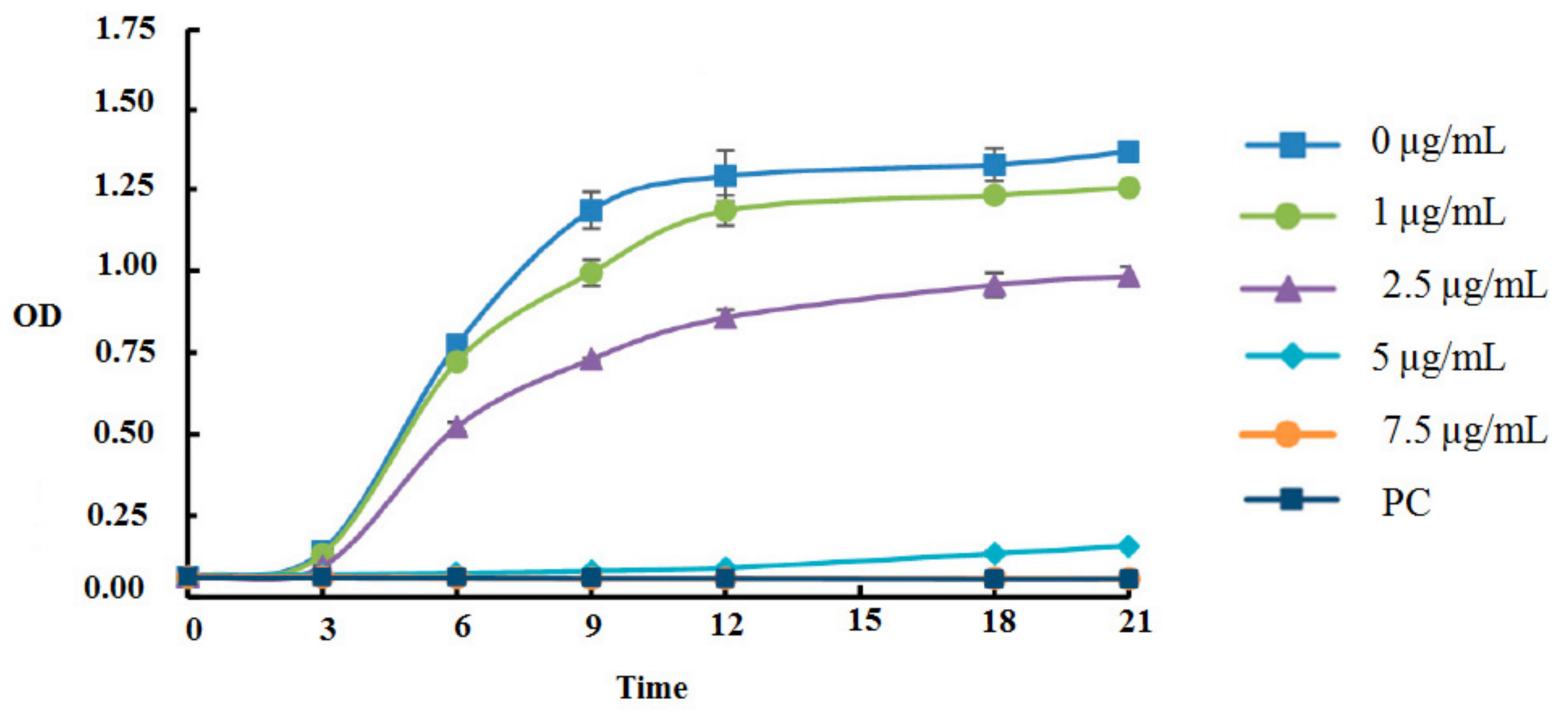
Assessment of time-kill kinetics of *A. baumannii* exposed to Secapin (1.0, 2.5, 5.0, and 7.5 µg/mL) at 3-hour intervals through optical density measurement at 595 nm (OD595)

In the time-kill kinetic assay, Secapin exhibited concentration- and time-dependent inhibition of bacterial growth (Figure 1 (Fig. 1)). In the negative control and at Secapin concentrations below the minimum inhibitory concentration (MIC), *A. baumannii* exhibited sigmoidal growth (typical growth curve) up to 21 hours, reaching the highest optical density (OD595) value in the control, followed by a gradual decrease in OD595 values with increasing concentrations. At the MIC, clear inhibition was observed for up to 12 hours, with a slight decrease in inhibition thereafter. When the Secapin concentration exceeded the MIC (7.5 µg/mL), bacterial growth was markedly suppressed, similar to the growth inhibition observed with chloramphenicol (50 µg/mL). 

The percentage of bacterial viability showed a concentration dependent response to Secapin (0–10 µg/mL) (Figure 1 (Fig. 1)). The highest viability was observed in the negative control (100%). At the MIC and MBC of Secapin, *A. baumannii* viability was significantly reduced (p<0.05) to 46% and 20.28% respectively. Furthermore, the observed values were similar to those obtained after treatment with chloramphenicol (50 µg/ml), where viability reached 17.6%.

### Secapin’s biofilm inhibition and eradication capabilities

**Figure 2:**
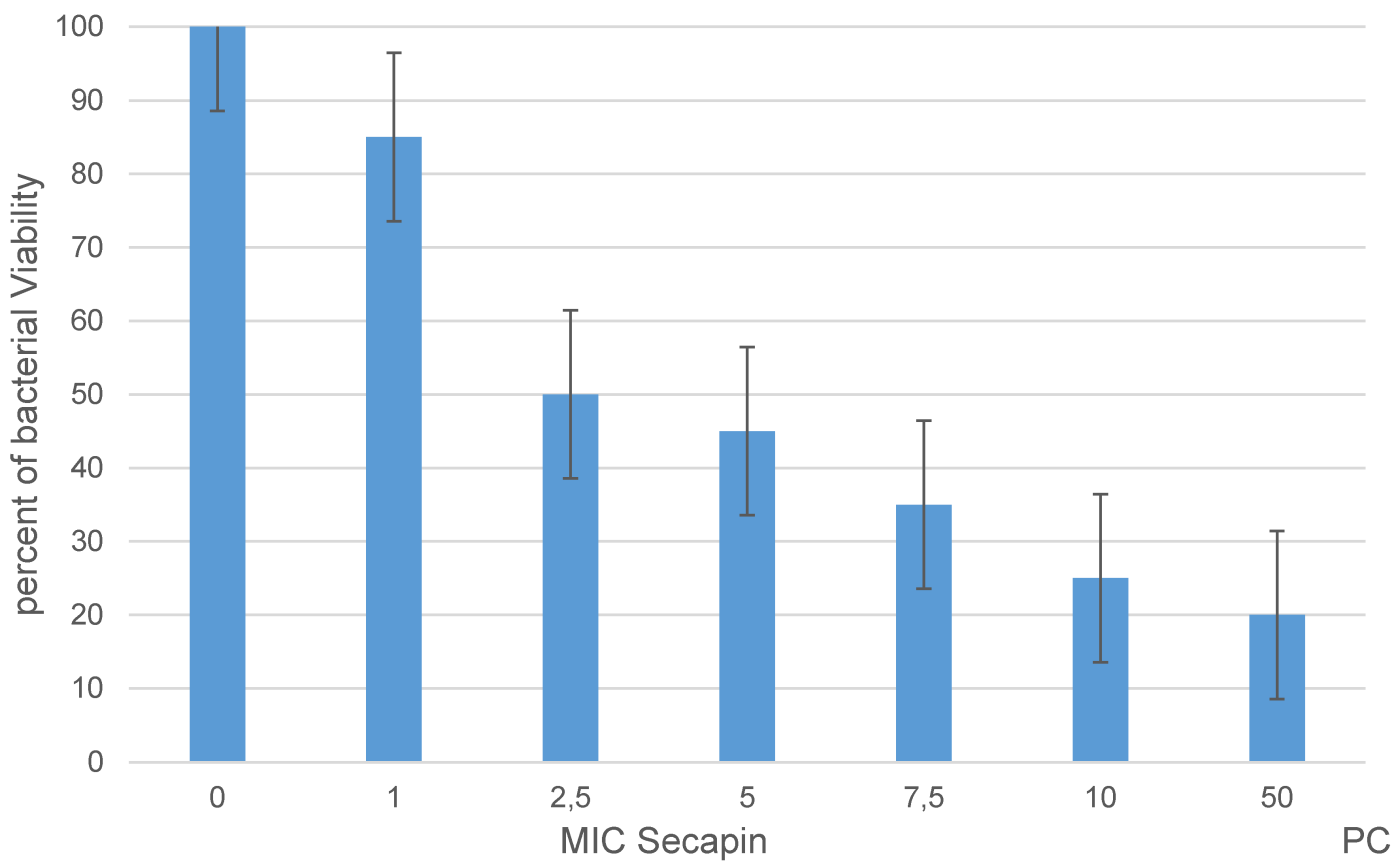
Viability of *A. baumannii* following Secapin treatment

**Figure 3:**
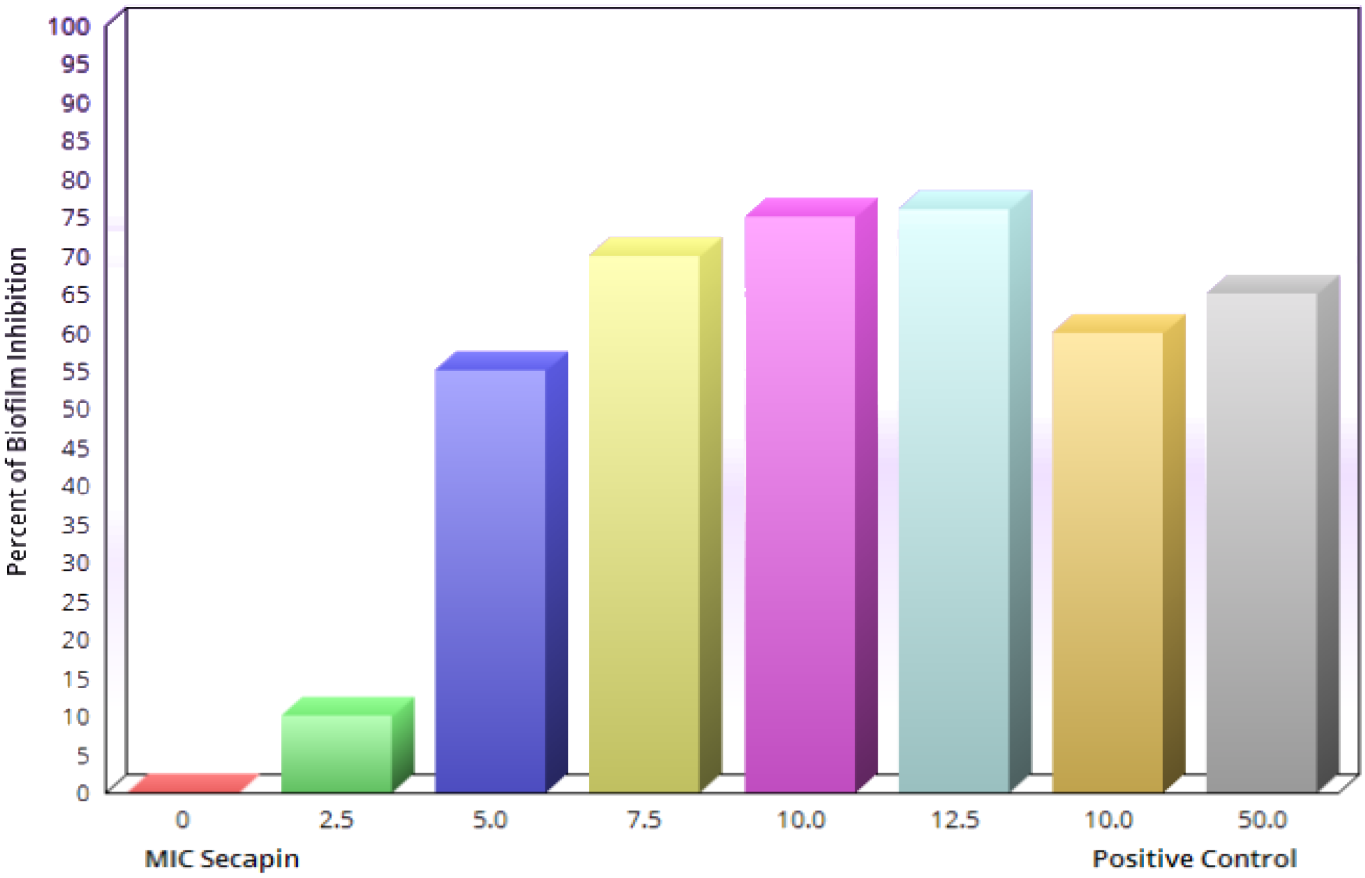
Quantitative assessment of biofilm formation inhibition by Secapin against *A. baumannii*

The inhibition and eradication of biofilm formation following treatment with Secapin is shown in Figure 2 (Fig. 2). Minimal biofilm inhibition was observed at 2.5 µg/mL Secapin, followed by a concentration-dependent inhibition trend of Secapin (Figure 3 (Fig. 3)). At the MIC (5 µg/mL) and MBC (10 µg/mL) of Secapin, the inhibition increased significantly (p<0.05) by 61.59% and 76.29%, respectively, compared to the negative control. In contrast, chloramphenicol at MBC (50 µg/mL) showed a lower inhibition (71.45%) than Secapin at MBC. 

**Figure 4:**
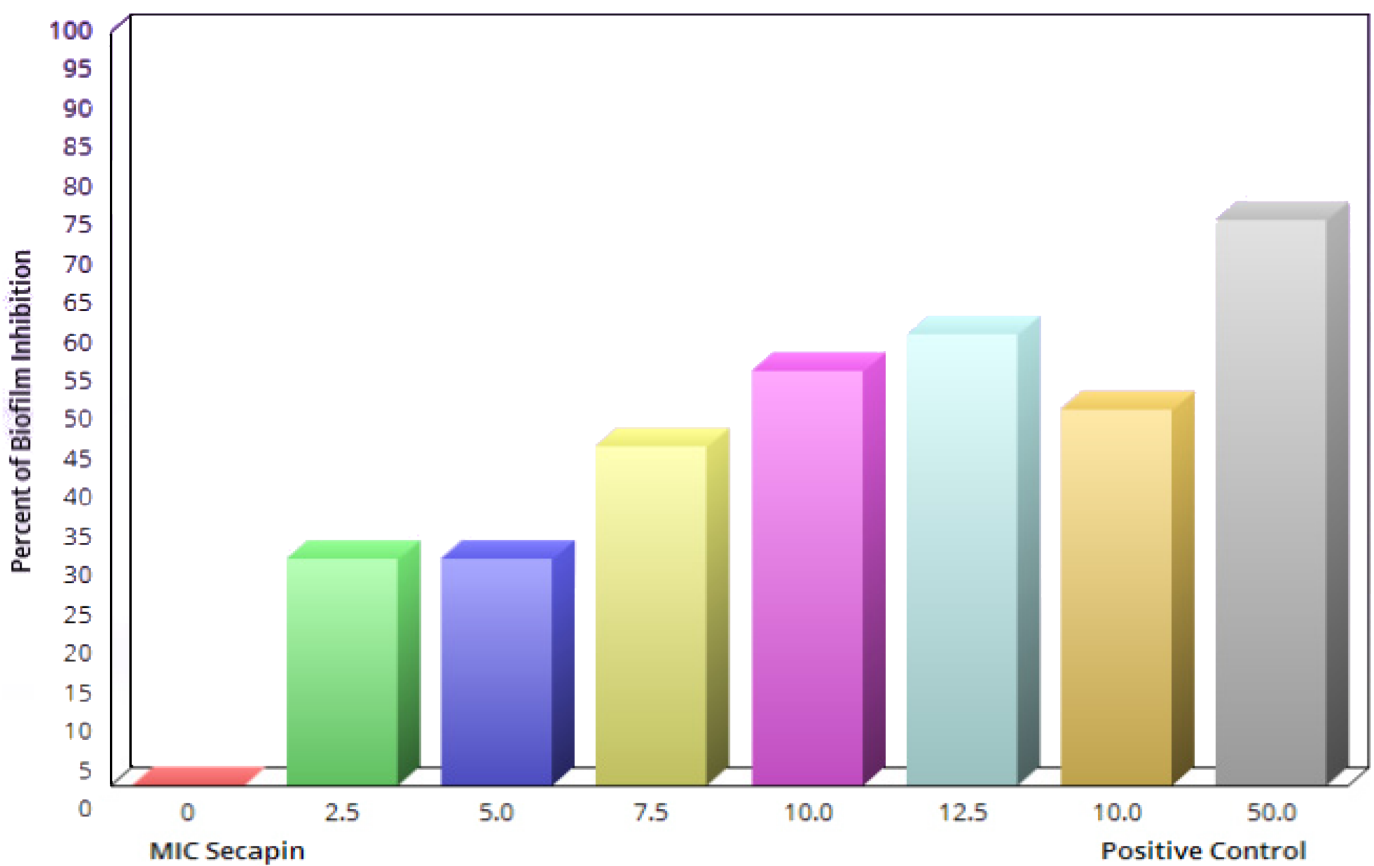
Quantification of *A. baumannii* biofilm eradication by Secapin

Similarly, the biofilm eradication results showed a concentration dependent eradication effect of Secapin (Figure 4 (Fig. 4)). The results indicated that preformed biofilms were significantly (p<0.05) eradicated by 35.62% and 53.19% at the MIC (5 µg/mL) and MBC (10 µg/mL) of Secapin, respectively. In contrast, chloramphenicol at MBC (50 µg/mL) resulted in a significantly higher eradication (76.19%) of preformed biofilms compared to the negative control.

### Hemolysis activity and cytotoxicity of Secapin

**Figure 5:**
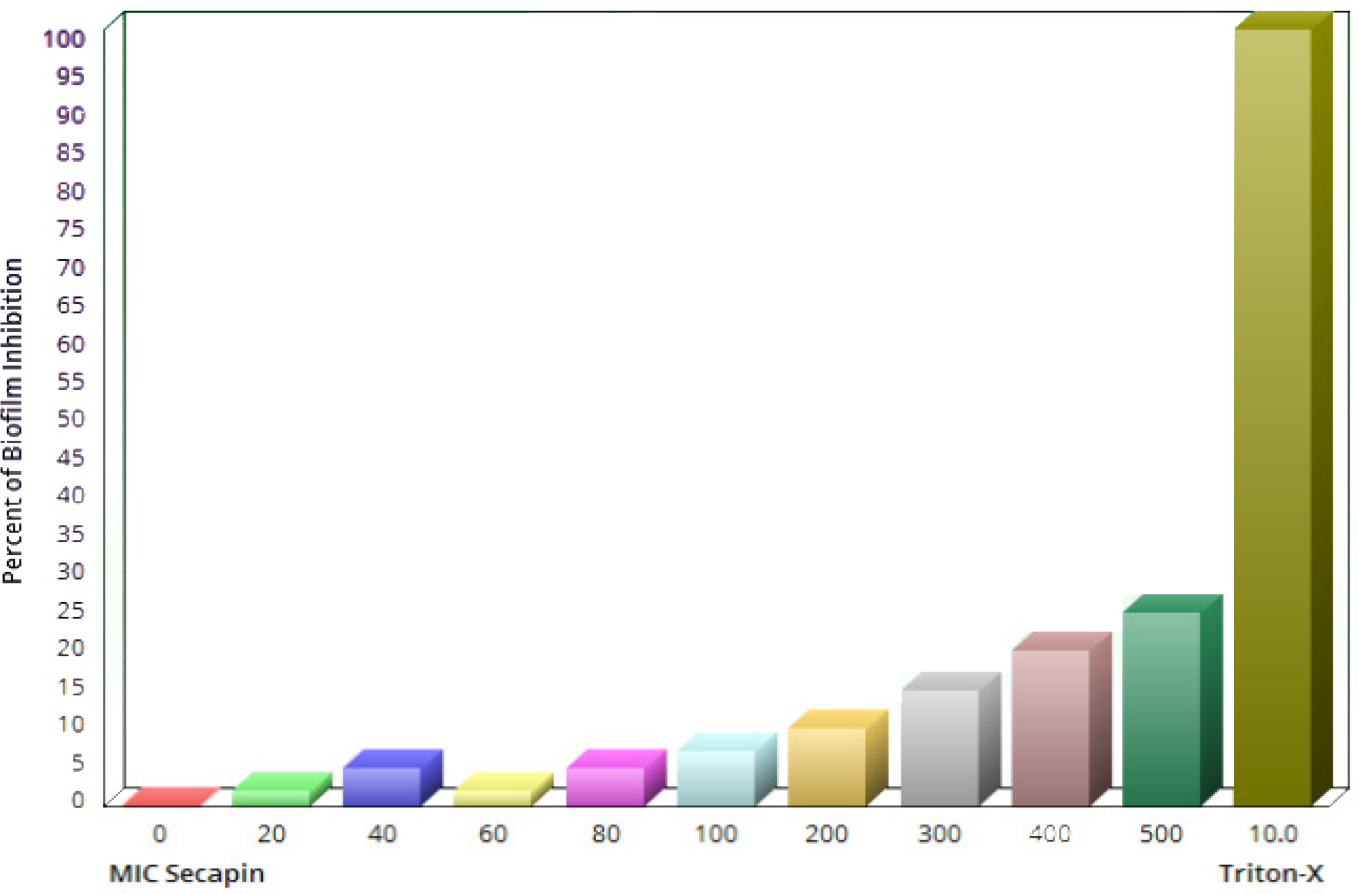
Quantification of hemolytic activity and cytotoxicity induced by Secapin on mouse red blood cells (RBC)

As shown in Figure 5 (Fig. 5), Secapin exhibited minimal haemolysis up to a concentration of 100 µg/mL, comparable to the negative control. However, an escalation in haemolysis was observed with increasing concentrations of Secapin. The peak haemolysis of 31.50% was observed at the highest concentration tested of 500 µg/mL, significantly lower (p<0.05) than the positive control (100%).

**Figure 6:**
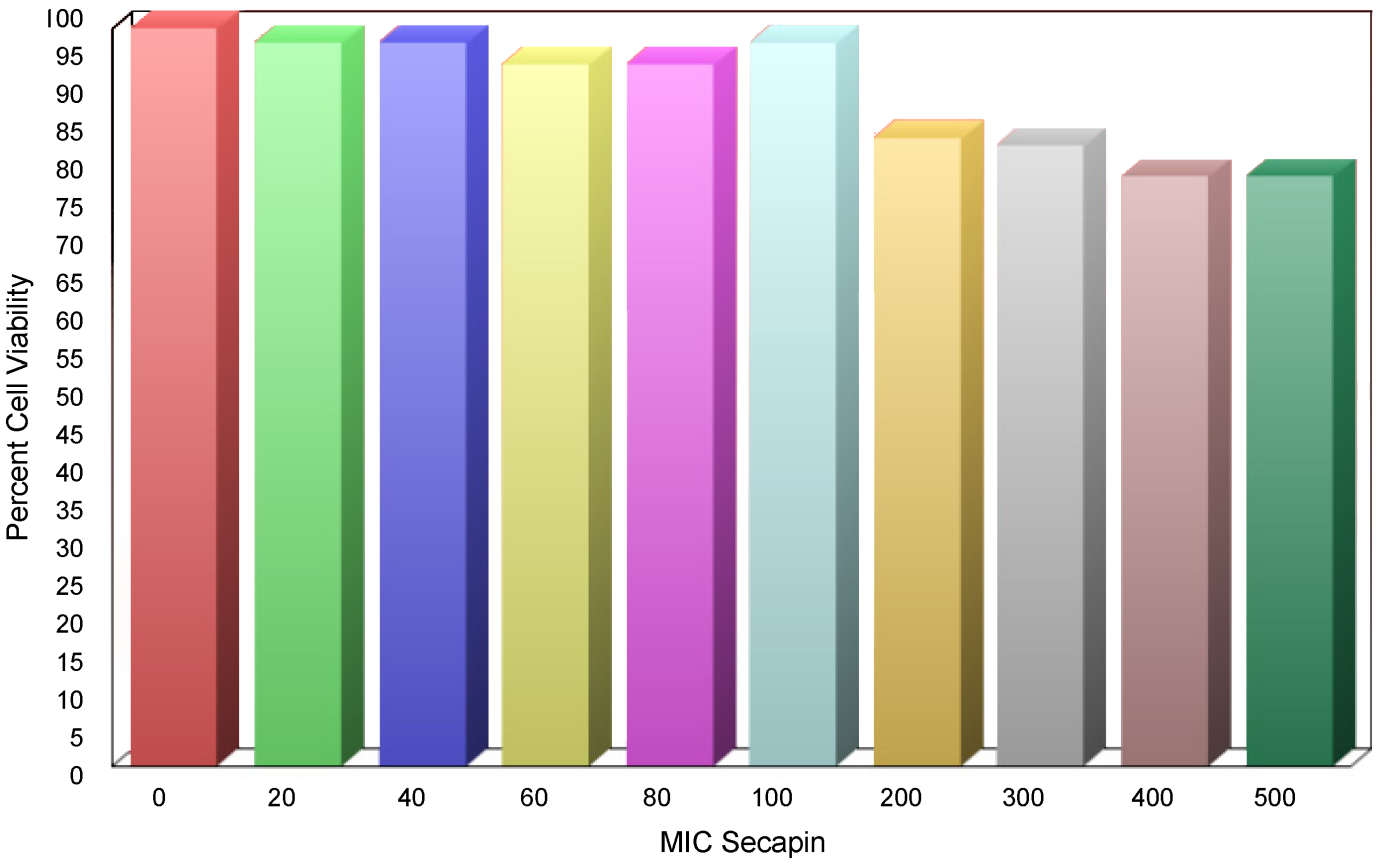
Evaluation of Secapincytotoxicity on murine raw 264.7 macrophage cells via MTT assay

Figure 6 (Fig. 6) shows the cytotoxicity evaluation of Secapin in RAW 264.7 cells. RAW264.7 macrophages treated with different concentrations of Secapin showed no significant (p>0.05) changes in cell viability up to 100 µg/mL compared to the negative control. At concentrations of 200 µg/mL and above, a significant (p<0.05) decrease in cell viability was observed, ranging from 86.53% to 80.70% compared to the negative control. It is noteworthy that in addition to changes in cell viability, no morphological changes were observed in the cells at the concentrations tested.

## Discussion

Antimicrobial peptides (AMPs) are pivotal components of innate immunity, serving as the initial barrier against pathogenic challenges [11]. Their swift bactericidal impact, extensive range of activity, diverse mode of action and minimal potential for resistance emergence have positioned both natural and synthetic AMPs at the vanguard of antimicrobial therapies [12]. This development marks the onset of a new era in addressing multidrug-resistant pathogens. Given the increasing prevalence of microbial resistance to conventional antibiotics, there is a growing emphasis on innovative therapeutic strategies targeting the treatment of MDR strains and nosocomial *Acinetobacter baumannii* infections [13].

In this study, a comprehensive analysis was conducted to evaluate the antibiotic resistance profiles of an *A. baumannii* strain, coupled with an investigation into the efficacy of Secapin in inhibiting bacterial growth and biofilm formation. Our findings revealed a significant resistance of *A. baumannii* strains to a wide range of antibiotics, encompassing aminoglycosides, glycopeptides, macrolides, cephalosporins, penicillin, lincosamide, diaminopyrimidine, and rifamycin. Only 22.2% of the strains demonstrated susceptibility to the tested antibiotics.

Consistent with our results, Son et al. [14] documented a similar antimicrobial resistance profile in an *A. baumannii* strain isolated from a patient with community-acquired pneumonia, demonstrating susceptibility to four antibiotics (colistin, minocycline, Gentamicin and tigecycline) out of ten major antibiotics tested. The emergence of multidrug resistance in *A. baumannii* can be attributed to various mechanisms including the presence of antibiotic-inactivating enzymes (β-lactamases, oxacillinases, and carbapenemases), active efflux systems, and inherent bacterial resistance due to restricted membrane permeability [15].

Our findings underscore the presence of extensive antibiotic resistance characteristics in the recently identified *A. baumannii* strain (hospitalized strain 37), attributed to the acquisition of various antimicrobial resistance factors. This underscores the urgent need for novel antibacterial agents to tackle multidrug-resistant *A. baumannii* and mitigate the escalating global health risks associated with the emergence of drug-resistant bacterial strains [16].

Our investigation delved into the intrinsic antibacterial attributes of the Secapin peptide sequence, focusing on its efficacy against A. baumannii. Our results unveiled that Secapin demonstrated potent growth inhibition and bactericidal effects on *A. baumannii* at remarkably low concentrations, with a MIC of 5 µg/mL and a MBC of 10 µg/mL, resulting in an MBC/MIC ratio of 2.0. Notably, the Secapin-treated groups exhibited lower MIC values compared to the chloramphenicol-treated groups, which displayed MIC and MBC values of 10 µg/mL and 50 µg/mL. Prior investigations have examined diverse antimicrobial peptides (AMPs) like LL-37 (MIC: 32 µg/mL; MBC: 128 µg/mL) [17] and omiganan, a synthetic antimicrobial peptide (MIC: 32 µg/mL; MBC: 64 µg/mL), targeting *A. baumannii* [18]. Our research demonstrates that Secapin showcases superior antibacterial efficacy with reduced MIC and MBC values, surpassing the effectiveness of the conventional antibiotic chloramphenicol. Additionally, in line with the criteria established by Traczewskiet al. [19], the low MBC/MIC ratio signifies the potent bactericidal activity of Secapin rather than a bacteriostatic effect. Time-dependent studies on bacterial killing and tests on bacterial survival further confirmed the dose-related ability of Secapin to kill *A. baumannii*. The increased efficacy of Secapin can be ascribed to its chemical properties, which facilitate its interaction with negatively charged molecules present on the surface of A. baumannii, thereby enabling efficient membrane penetration and providing insight into its bactericidal mechanism.

The issue presented by antibiotic-resistant bacteria, such as *A. baumannii*, is further complicated by the emergence of biofilms. These biofilms enhance bacterial attachment to surfaces through the creation of intricate structures consisting of diverse microorganisms and extracellular polymeric substances, such as exopolysaccharides, proteins, lipids, and DNA [20]. The limited penetration of antibiotics through biofilms, coupled with antibiotic entrapment, enzymatic degradation within biofilms, and reduced metabolic activity in the basal biofilm layer, underscores the critical need to control biofilm-forming bacteria [21]. Antimicrobial peptides (AMPs) hold promise as antibiofilm agents by impeding initial bacterial growth and eradicating mature biofilms [22]. Notably, Peng et al. highlighted the potent biofilm inhibition and eradication capabilities of the peptide Cec4 against clinical *A. baumannii* isolates, achieving a significant reduction in biofilm formation with low peptide concentrations [23]. Similarly, our study revealed that Secapin effectively suppressed *A. baumannii* biofilm formation by over 75% at the MBC of 10 µg/mL, surpassing the efficacy of chloramphenicol at 50 µg/mL. This suggests that Secapin functions as an antibiofilm agent, likely through its bactericidal activity, disrupting *A. baumannii* biofilm development. Furthermore, our results demonstrated Secapin’s ability to eradicate established biofilms by 53.19% at the MBC level, comparable to chloramphenicol at the same concentration. These findings underscore Secapin’s robust antibiofilm activity against MDR* A. baumannii* strains, possibly attributed to its ability to penetrate the biofilm matrix. However, we observed relatively lower antibiofilm efficacy of Secapin (approximately 50% remaining biofilm at 10 µg/mL) against preformed biofilms compared to biofilm formation inhibition, highlighting the greater challenge posed by disrupting preexisting biofilms. Consistent with these observations, Kim et al. [10] reported residual biofilm following biofilm eradication assays with magainin 2 against *A. baumannii*. Collectively, our data support Secapin’s efficacy in inhibiting biofilm formation and effectively disrupting preformed biofilms, positioning it as a promising candidate for developing antimicrobial agents against MDR *A. baumannii*.

When contemplating the use of antimicrobial peptides (AMPs) for therapeutic purposes, their potential harm to mammalian cells poses a significant barrier to the progression of these compounds into clinical trials or commercialization for human use. The assessment of AMP cytotoxicity commonly relies on hemolysis and lymphocyte cell viability assays. In our study, Secapin displayed minimal hemolytic effects, showing almost undetectable hemolysis at both the MIC of 5 µg/mL and the MBC of 10 µg/mL. Remarkably, even at higher concentrations (500 µg/mL), Secapin exhibited low hemolytic activity, measuring at 31.50%.

## Conclusion

The findings of our research definitively establish the potent antimicrobial efficacy of Secapin against MDR strains of *A. baumannii*. The remarkable ability of the peptide to impede bacterial proliferation, along with its favorable safety profile, positions it as a compelling candidate for progressive exploration as a therapeutic modality against *A. baumannii* infections. Subsequent investigations should be directed towards elucidating the intricate mechanisms governing Secapin antimicrobial activity and refining its attributes to enhance its efficacy in clinical settings for addressing the challenge posed by MDR bacterial strains.

## Limitation

The limitation of this study was that we worked on a strain of *A. baumannii* and did not have the financial resources to work on more resistant strains.

## Notes

### Authors’ ORCID

- Mahsa Kavousi: 0000-0002-1635-743X
- Mahnaz Farahmand: 0000-0002-7535-2217

### Competing interests

The authors declare that they have no competing interests.

## Figures and Tables

**Table 1:**
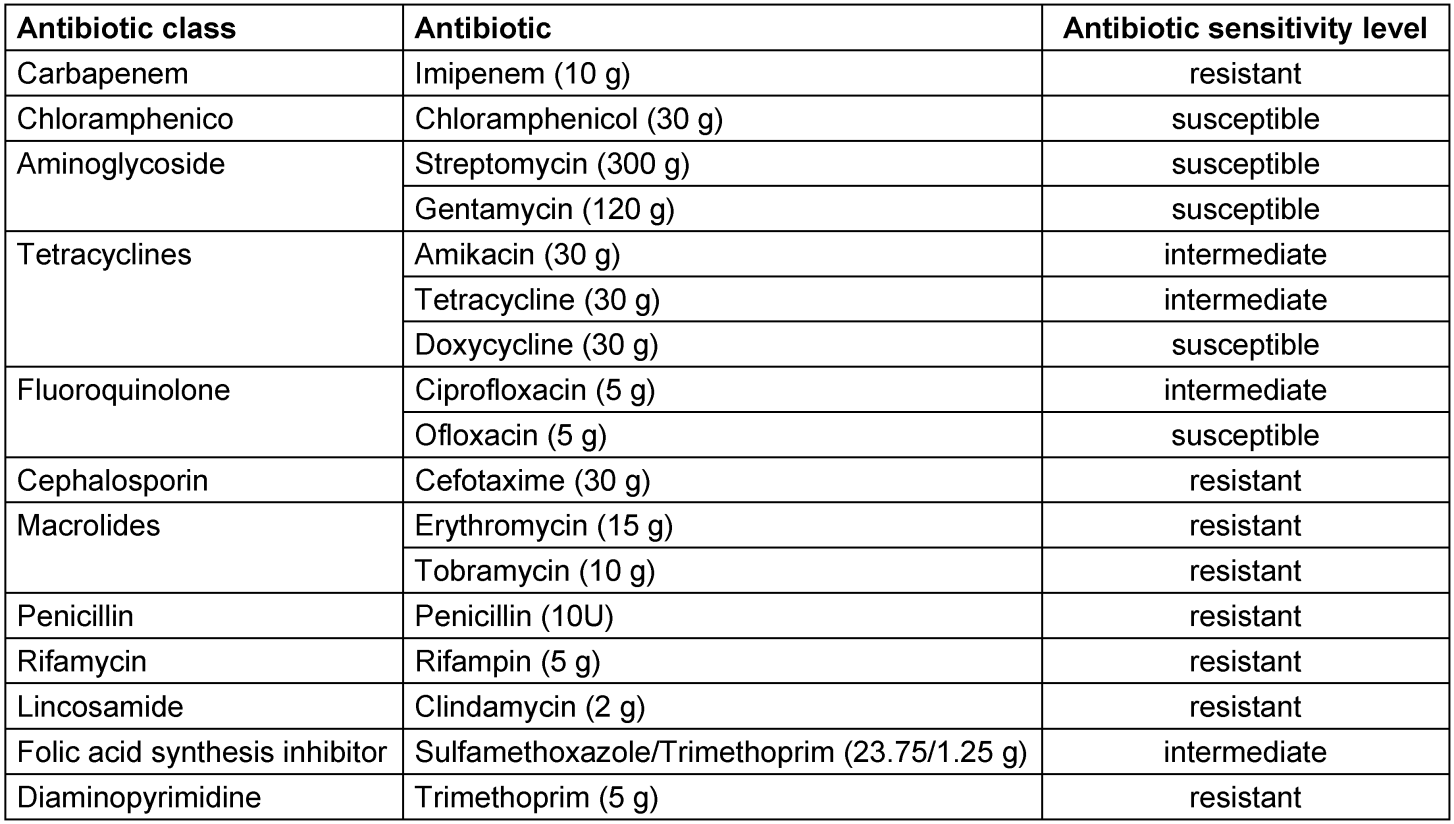
Antibiotic sensitivity pattern of *A. baumannii* (hospitalized strain 37)
